# Preliminary Evaluation of the Engagement and Effectiveness of a Mental Health Chatbot

**DOI:** 10.3389/fdgth.2020.576361

**Published:** 2020-11-30

**Authors:** Kate Daley, Ines Hungerbuehler, Kate Cavanagh, Heloísa Garcia Claro, Paul Alan Swinton, Michael Kapps

**Affiliations:** ^1^Vitalk, TNH Health, São Paulo, Brazil; ^2^School of Psychology, University of Sussex, Brighton, United Kingdom; ^3^Department of Preventive Medicine, Medical School, University of São Paulo, São Paulo, Brazil; ^4^School of Nursing, University of Campinas, Campinas, Brazil; ^5^School of Health Sciences, Robert Gordon University, Aberdeen, United Kingdom

**Keywords:** digital health, chatbot, conversational agent(s), mental health, depression, anxiety, stress, low-and middle-income

## Abstract

**Background:** Mental health difficulties are highly prevalent, yet access to support is limited by barriers of stigma, cost, and availability. These issues are even more prevalent in low- and middle-income countries, and digital technology is one potential way to overcome these barriers. Digital mental health interventions are effective but often struggle with low engagement rates, particularly in the absence of human support. Chatbots could offer a scalable solution, simulating human support at a lower cost.

**Objective:** To complete a preliminary evaluation of engagement and effectiveness of Vitalk, a mental health chatbot, at reducing anxiety, depression and stress.

**Methods:** Real world data was analyzed from 3,629 Vitalk users who had completed the first phase of a Vitalk program (“less anxiety,” “less stress” or “better mood”). Programs were delivered through written conversation with a chatbot. Engagement was calculated from the number of responses sent to the chatbot divided by days in the program.

**Results:** Users sent an average of 8.17 responses per day. For all three programs, target outcome scores reduced between baseline and follow up with large effect sizes for anxiety (Cohen's d = −0.85), depression (Cohen's d = −0.91) and stress (Cohen's d = −0.81). Increased engagement resulted in improved post-intervention values for anxiety and depression.

**Conclusion:** This study highlights a chatbot's potential to reduce mental health symptoms in the general population within Brazil. While findings show promise, further research is required.

## Introduction

Mental health disorders are highly prevalent, yet numbers accessing treatment are low (1, 2). Reasons cited for this discrepancy include structural barriers such as a lack of availability, high cost and attitudinal barriers such as perceived stigma (3, 4). Advances in digital technology along with increases in internet access and smartphone ownership could offer an opportunity to overcome some of these barriers, bringing relative anonymity and scalability at a lower cost (5, 6).

There is growing empirical support for use of digital technology in mental health, where web platforms or smartphone applications are used to deliver evidence-based interventions. These digital interventions are effective, feasible and acceptable to users (7–9), although are associated with low engagement and poor adherence (10, 11). Adherence rates and outcomes appear more favorable when active human support or guidance is integrated into the digital tool (12, 13), but this limits scalability. The development of conversational agents, or chatbots could offer an interesting solution (14, 15). Chatbots take a conversational approach, simulating human interaction through written text. This ability to mimic human support could potentially improve engagement while enabling scalability through automation. This could be particularly interesting in low- and middle- income settings where there are large mental health treatment gaps and scarce resources available (6).

Within mental health, chatbots have been used to deliver psychoeducation, self care strategies and skills training and show high satisfaction rates (14, 15). The use of chatbots appears feasible and acceptable to users (15, 16), but there is less certainty on effectiveness. The knowledge base is however growing rapidly, with recent studies finding that engaging with a conversational agent over relatively short periods can reduce perceived stress (11) and improve symptoms of depression (5) and anxiety (5, 17). These studies are all RCTs or pilot RCT's and challenges surrounding user engagement means that real world uptake may differ (10). Additionally, the studies were all completed in high income countries, with small sample sizes (*n* < 75) and recruited from student populations.

To our knowledge there have been no studies to evaluate the use of mental health chatbots within Latin America. This study seeks to explore if a mental health chatbot has the potential to be effective within Brazil.

## Aim

To evaluate real world engagement and effectiveness of Vitalk, a newly developed mental health chatbot. The hypothesis is that use of the chatbot will lead to a reduction in symptoms of stress, anxiety and depression over a 1 month period.

## Methods

### Participants

Vitalk was installed voluntarily by members of the general population who had found it on the hosting platform, through marketing campaigns, word of mouth, or a personal search. Vitalk sought to offer conversations about mental health and self help strategies to improve well-being. Users had installed Vitalk between June and November 2019 and completed 1 month of a Vitalk program (*N* = 3,629). All users were Portuguese speakers located in Brazil and over 18 years of age, with internet access.

### Description of Chatbot

Vitalk is an automated chatbot delivering mental health content in an innovative conversational format. It is a free-to-use service, hosted within an instant messenger platform, accessible from any internet-enabled device. The chatbot is built on ruby and JavaScript and was created by the IT, product development and UX team at TNH Health.

The overall aim of Vitalk is to improve well-being by reducing stress, anxiety and depression using a preventative approach to mental health. It consists of three core programs, addressing themes of anxiety, low mood, and stress. Programs are 90 days in duration, divided into three 30-day phases. During each phase, the user is engaged in four to five conversations per week, each lasting ~5 min. Outcome measurement and feedback on scores is built into the program, with users completing symptom questionnaires at baseline and the end of each phase.

The conversations are based on insights and strategies taken from Cognitive Behavioral Therapy (CBT) and Positive Psychology (18, 19), which include (but are not limited to) psychoeducation, cognitive restructuring, behavioral activation, gratitude, and practical exercises such as breathing, relaxation and meditation. The goal of the conversations is to help the user reflect on experiences and learn techniques which can help them manage stress, mood and anxiety. A mood tracking tool helps to aid reflection, “emojis” and GIFs to mimic a natural human interaction and gamification to increase engagement. The chatbot avatar is named Viki and was developed through focus groups and piloting with users. The conversations used by Viki are written by a team of Clinical Psychologists and healthcare professionals and shaped by a user experience (UX) team who work on the style and design of the chatbot. This involves adapting including the words language used to make the language more accessible to the user.

**Figure 1:**
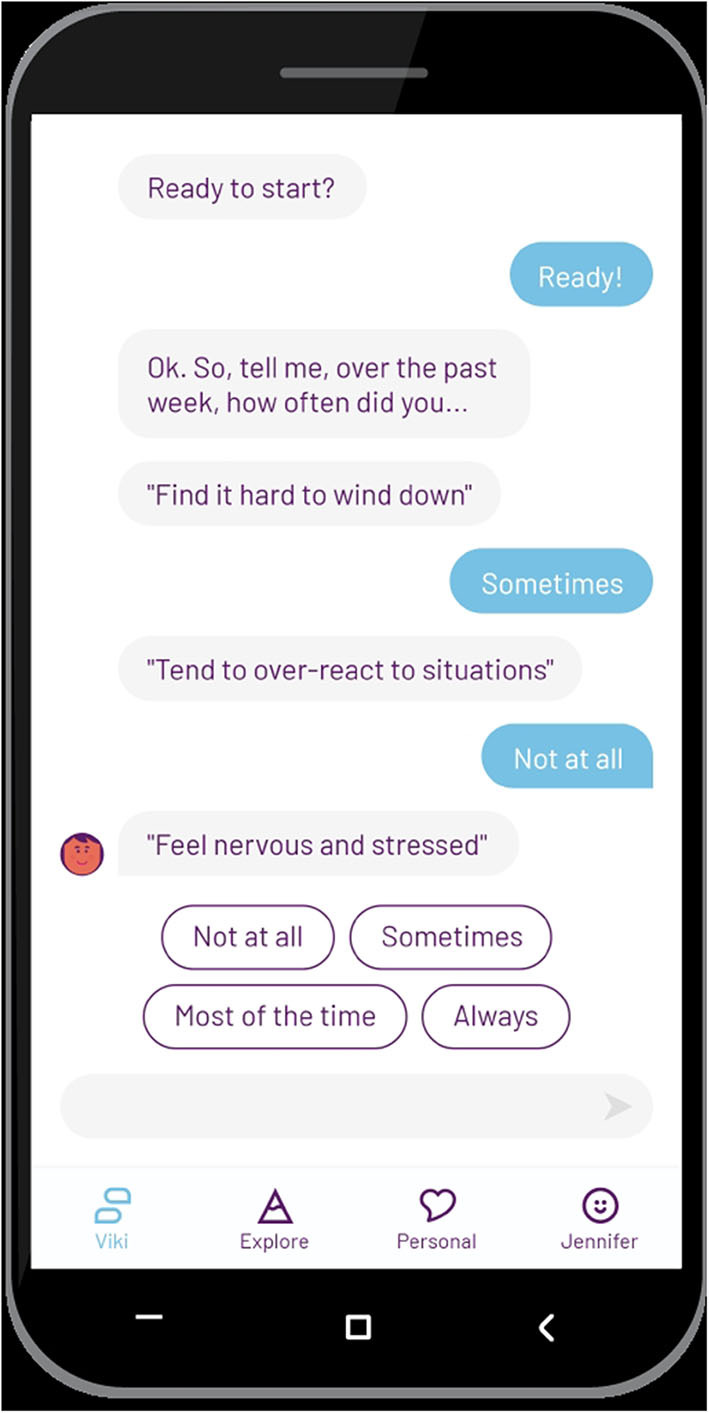
Screenshot of Vitalk check-up.

The chatbot initiates conversations, and the user can reply by picking a predefined answer option (Figure 1). The predefined options customize the conversation to suit user needs and preferences. In some cases, the user can also enter free text, for example between conversations or if they want to interrupt the flow. Natural language understanding (NLU) triggers conversational modules based on keywords identified from the text entered by the user, which then loops back to the pre-programmed conversations. As an example of free text, if the user types “good morning” the greetings module is triggered and the chatbot will respond to the greeting, ask how the user is feeling and offer a suitable activity. In a situation where the user types words directly or indirectly linked to suicidality such as “life isn't worth living,” the risk module is triggered and the chatbot delivers crisis information. This is continuously refined and adapted to ensure the language and emotional expression matches that of the user.

Vitalk does not aim to replace a healthcare professional or to offer treatment. Users are made aware of this in the terms and conditions they consent to, and they are advised to seek additional support if they show a high risk of depression or anxiety during the assessment. Where the system identifies a risk issue, the user is sent details of support services, including the national suicide line, and where appropriate, a follow up conversation with a healthcare professional from Vitalk is initiated.

## Measures

### Generalized Anxiety Disorder (GAD-7)

The GAD-7 is a 7-item self-report scale used to assess anxiety symptoms over the past 2 weeks *(e.g., how often have you been bothered by feeling afraid something awful might happen)*. Scores range from 0 (not at all) to 3 (nearly every day) with a total of 21. The total scores are divided into four categories: none (0–4), mild (5–9), moderate (10–14) and severe (15+) symptoms (20, 21).

### The Patient Health Questionnaire (PHQ-9)

The PHQ-9 is a 9 item self-report scale that evaluates symptoms of depression over the past 2 weeks (*e.g., how often have you been bothered by feeling down, depressed, or hopeless)*. Item response options use a Likert scale ranging from 0 (not at all) to 3 (nearly every day). Total scores are divided into five categories: none (0–4), mild (5–9), moderate (10–14), moderately severe (15–19) and severe (20+) symptoms (22, 23).

### Depression, Anxiety and Stress Scale (DASS-21) - Stress Subscale

The DASS-21 is a 21 item self-report questionnaire consisting of three scales to measure depression, anxiety, and stress. Only the stress subscale was used, this consists of seven items, and the user is asked how much each statement applies to them in the past week (*e.g., I found it difficult to relax)*. Scores range from 0 (did not apply) to 3 (applied very much or most of the time). Total scores are doubled and divided into five categories: normal (0–14), mild (15–18), moderate (19–25), severe (26–33) and extremely severe (34+) levels of stress (24, 25).

All scales have been translated into Brazilian Portuguese and are validated for use within this population.

## Procedures

After installing Vitalk, users complete a sign-up process that includes consenting to their anonymized data being used for research purposes. The user is then engaged in a conversation with the chatbot, who welcomes them to Vitalk and together they complete the baseline outcome measures. User responses are recorded and scored by the chatbot, and the user receives customized feedback based on their scores. The user then chooses which of the three programs to complete, or they can ask the chatbot to recommend one based on their scores.

After a program has been selected, the core content is scheduled for delivery to the user over the course of 30 days. When new content is delivered, the user is alerted to a new message, and they choose whether to engage in the conversation. The responses of the user shape the conversation. Original content is delivered on the next scheduled day, regardless of previous engagement. The user can discontinue the program or close their account at any time.

At the end of phase one (day 30), the outcome measure corresponding to the active program is repeated (GAD-7 for the anxiety program, PHQ-9 for low mood, DASS-21 for stress). At this point, the user can continue with the program they are in or swap to another program if their goal has shifted. A full check-up consisting of all three measures is repeated at the end of the program (day 90).

For this study, we explored results from the first phase of each program to ascertain early engagement and explore initial effects.

### Data Collection and Privacy

The study involved real-world data obtained from an anonymous, non-clinical population. The users agreed to the terms of service and privacy policy when they signed up, which includes giving consent for their anonymized data to be used for research purposes. Access to the chatbot requires a login and is password protected, and data gathered by Vitalk is stored on a secure server. Data were extracted from the server, anonymized and provided to the authors for data analysis. Demographic data was collected by the hosting platform who provided this to TNH Health in an aggregated format.

### Statistical Analysis

Three sets of univariate analyses were conducted with data collected from the GAD-7, PHQ-9 and DASS-21 stress scales. Engagement with Vitalk was quantified as a rate measure by dividing the number of messages sent by the number of days in the program. Descriptive statistics were used to describe centrality and distribution of pre-post scale values, severity of symptoms and engagement. To describe the magnitude of change across the intervention, average based change was quantified with Cohen's d and standard reference values (0.2: “small,” 0.5: “medium,” and ≥0.8: “large” used to qualitatively describe the effect) (26).


$$\begin{array}{l} d=\left(M_{post}-M_{pre}\right)/\;S_{diff} \end{array}$$

Where *M* is the mean and *S*_*diff*_ is the standard deviation of the change in scale values across the intervention. For the PHQ-9 and GAD-7 scales, reliable change and clinical caseness was assessed. For each scale, the Reliable Change Index was calculated by multiplying the standard error of the difference by 1.96 (27).


$$\begin{array}{l} SE_{diff}=S_{pre}\sqrt{2}\sqrt{1-r} \end{array}$$

*S*_*pre*_ is the standard deviation of pre-intervention values and *r* is the reliability obtained in validation studies of each scale (28). To be considered reliable, change in scale values across the intervention had to exceed 5.9 for the PHQ-9 and 3.7 for the GAD-7.

Clinical caseness was examined by calculating the number of people who were above the clinical cut off at baseline and moved below this cut off at follow-up. The number of these people that experienced a reliable change was also quantified. A cut-off score of ≥10 was used for the PHQ-9, and ≥8 for the GAD-7.

Modeling of post scale values were completed with pre scale values and engagement included as predictor variables. Because the scales used can exhibit floor and ceiling effects with many observations at the upper value, tobit regression models were used to deal with the truncated nature of the data and limit biased estimates that can be obtained with Ordinary Least Squares Regression (29). All statistical analyses were conducted in R version 3.5.3 (30).

## Results

In total, 3,629 users were included in this study. They were located across Brazil; the majority were female (76%) and aged 18–24 years (52%) (536/1,648, 32.5%).

### Symptom Change

#### Less Anxiety Program

In total, 1,648 (45.4%) users completed the first phase of the anxiety program.

The average anxiety score (GAD-7) reduced from moderate anxiety (Table 1) (mean 12.2, SD = 4.7) to mild anxiety (mean 8.1, SD = 4.7). This reduction was −4.1 ± 4.8 [*t*_(1, 647)_ = −34.3, *p* < 0.001) with Cohen's d = −0.85 indicating a large effect. The percentage of reliable improvement and reliable decline was equal to 52.2 and 4.7%, respectively. In terms of clinical caseness, 655 (49.0%) users who were above the clinical cut-off at baseline moved to below clinical cut-off at follow up [GAD-7 ≥ 8), 557 (41.6%) of whom also showed reliable improvement].

**Table 1:** Classification of anxiety symptoms pre- and post-intervention (% of participants in each classification using GAD-7).

	**None**	**Mild**	**Moderate**	**Severe**
Pre	58 (3.5%)	484 (29.4%)	570 (34.6%)	536 (32.5%)
Post	382 (23.2%)	764 (46.4%)	311 (18.9%)	191 (11.6%)

#### Better Mood Program

In total 1,243 users (34.3%) completed the first phase of the depression program.

The average depression score (PHQ-9) reduced from a moderately severe level (mean 15.9, SD = 6.5) to a moderate level (mean 10.4, SD = 6.5) (Table 2). This reduction was −5.5 ± 6.0 [*t*_(1, 242)_ = −31.9, *p* < 0.001) with Cohen's d = −0.91 indicating a large effect. The percentage of reliable improvement and reliable decline was equal to 45.1 and 2.3%, respectively. In terms of clinical caseness 449 (46.3%) of users who were above the clinical cut-off at baseline moved to below clinical cut-off at follow up (PHQ-9 total score ≥10), of whom 370 (38.1%) also showed reliable improvement.

**Table 2:** Classification of depression symptoms pre- and post-intervention (% of participants in each classification using PHQ-9).

	**None**	**Mild**	**Moderate**	**Moderately Severe**	**Severe**
Pre	43 (3.5%)	230 (18.5%)	249 (20.0%)	297 (23.9%)	424 (34.1%)
Post	210 (16.9%)	480 (38.6%)	250 (20.1%)	156 (12.6%)	147 (11.8%)

#### Less Stress Program

In total, 738 users (20.34%) completed the first phase of the stress program.

Within this period, the average stress score (DASS-21 stress scale) was reduced from severe levels (mean 25.8, SD 9.1) to mild levels (mean 17.5, SD = 9.3) (Table 3). This reduction was −8.3 ± 10.3 [*t*_(737)_ = −21.9, *p* < 0.001] with Cohen's d = −0.81 indicating a large effect. The percentage of reliable improvement and reliable decline was equal to 60.3 and 9.2%, respectively.

**Table 3:** Classification of stress symptoms pre- and post-intervention (% of participants in each classification using DASS-21 stress scale).

	**Normal**	**Mild**	**Moderate**	**Severe**	**Extremely Severe**
Pre	113 (15.3%)	72 (9.8%)	138 (18.7%)	242 (32.8%)	173 (23.4%)
Post	349 (47.3%)	101 (13.7%)	122 (16.5%)	112 (15.2%)	54 (7.3%)

### Engagement

In terms of engagement, the average number of responses sent by users to Vitalk per day (total responses divided by days in program) within the first phase of the program was 8.17 (SD, 3.67), with a range of between 0.29 and 40.25 responses.

Tobit regression models were used to assess the effects of engagement on intervention effectiveness after controlling for pre-intervention values (Table 4). Increased engagement resulted in improved post-intervention values for anxiety and depression (*p* < 0.01) but not stress (*p* = 0.716). No interaction effect was identified between engagement and pre-intervention values for any of the outcomes measured (*p* ≥ 0.08).

**Table 4:** Tobit regression models quantifying the effect of pre-intervention values and engagement with Vitalk as predictors of post-intervention anxiety, depression and stress values.

	**Anxiety (0–21) GAD-7**	**Depression (0–27) PHQ-9**	**Stress (0–42) DASS-21**
**Base Model: No interaction**	**Standardized coefficients (β)**	**Standardized coefficients (β)**	**Standardized coefficients (β)**
Intercept	8.1^***^	10.4^***^	17.2^***^
Pre	2.2^***^	3.8^***^	3.4^***^
Engagement	−0.33^**^	−0.46^**^	0.11
**Full Model: With interaction**			
Intercept	8.1^***^	10.4^***^	17.2^***^
Pre	2.2^***^	3.7^***^	3.4^***^
Engagement	−0.33^**^	−0.45^**^	0.13
Interaction (Pre:Engagement)	−0.07	−0.26	−0.19

*Pre and engagement variables were standardized by subtracting mean and dividing by the standard deviation*.

*** *p < 0.001*,

** *p < 0.01*.

## Discussion

Initial results indicate that the use of a mental health chatbot within this population can engage users and significantly reduce symptoms of anxiety, depression and levels of stress. Higher engagement with the chatbot, as measured by the number of responses sent by the user was also found to predict lower anxiety and depressive symptoms at follow up. To our knowledge this is the first study to examine the use of a mental health chatbot in Latin America, and results appear promising.

The majority of the sample were adult females under 24 years of age (52% 18–24 years, 76% female). This differed somewhat to the typical demographic profile of the host platform (29% 25–34 years, 54% female) (31) but it is not clear if this reflects the chatbot appealing more to this demographic, differing mental health prevalence rates (32), levels of help seeking behavior (33) or marketing bias.

Baseline levels of anxiety and depression were significantly higher than would be expected in the general population in Brazil based on previous research using the same measures (34, 35). This was not unexpected given users had actively sought out a mental health tool but does indicate that Vitalk is able to successfully reach its target audience and could be a useful way to engage individuals experiencing elevated stress and mental health symptoms. This finding fits with the idea that people recruited through methods that imply more active treatment-seeking such as a Google or Facebook search, present with high levels of anxiety and depression than through more passive methods (36). Interestingly, those recruited by online advertisements have been found to have similar levels of depression and anxiety as those recruited from clinical settings, suggesting the chatbot could be a helpful way to widen access and identify those most at risk, facilitating access to appropriate professional support (36).

Reliable improvements were found in all three programs with symptoms of anxiety, depression and stress reducing between baseline and follow up with large effect sizes. These findings are in line with other chatbot studies (5, 17, 37) but with a much larger sample size and suggests applicability within Brazil. Interestingly, large numbers of users showed clinically significant, reliable change, moving from a clinical to non-clinical range for anxiety and depression as measured by the PHQ-9 and GAD-7. Whilst this requires follow-up and further investigation, these initial results are extremely promising. It is unclear why there is such a magnitude of change, but one hypothesis is that participants had independently sought out a mental health tool and so were likely to have high motivation and a readiness to change.

## Limitations

This study presents preliminary data obtained over a brief engagement period without a control group. While an early response is found to be a strong predictor of the treatment outcome (38), there is a need to complete a more rigorous study with a control group and longer follow up before drawing any firm conclusions. It is hoped that the evidence presented in this paper will direct further clinical research and help in the planning of new technological interventions within this population.

Within this study, we had limited information on the users of Vitalk due to the method of data collection within the host platform. The lack of individual-level demographics limited the analysis. In future work it would be important to obtain more detailed information and a deeper understanding of users, for example, individual-level demographics and data on education levels and socio-economic status which may impact outcomes. It would also be interesting to explore if the users have had any psychological intervention in the past and if they are doing so currently. This would offer insight into who Vitalk is reaching, and if for example it is being used as an adjunct or follow on from therapy or as the only intervention being accessed. This would offer interesting learning for the wider health system.

Due to the study being retrospective, we defined engagement level as the average daily number of responses sent by the user, as this was the best measure based on the type of data available. However, this did not consider that some conversations are longer than others and the user may not be active every day. Exploring alternative engagement metrics would be useful to enrich our understanding of how the chatbot is used and would enable more comparison within the literature. Analyzing engagement for each conversation would also allow a more granular understanding of the impact of specific intervention techniques on symptom change. This would allow us to explore which are the most impactful components, whether this be the chatbot, mood tracking, specific cognitive behavioral techniques or a combination of all three.

## Conclusion

As there is currently very little research on the use of chatbots in mental healthcare, particularly within low- and middle-income countries, these early findings are promising. The study highlights the potential of using such an innovative tool to engage users and offer an effective intervention to improve mental health in Brazil's general population. While findings show promise, more rigorous research is required before any firm conclusions can be drawn.

## Data Availability Statement

The data analyzed in this study is subject to the following licenses/restrictions: Access upon request due to confidential or commercially sensitive data sets. Data anonymized and for research purposes only. Requests to access these datasets should be directed to Ines Hungerbuehler, ines@tnh.health.

## Ethics Statement

Ethical review and approval was not required for the study on human participants in accordance with the local legislation and institutional requirements. Written informed consent for participation was not required for this study in accordance with the national legislation and the institutional requirements.

## Author Contributions

KD and IH contributed substantially to the conception and design of the study, the acquisition of data, and writing of the paper. KC and HC provided critical review and significant contribution to the manuscript. PS completed data analysis. MK contributed to initial study design. All authors contributed to the article and approved the submitted version.

## Conflict of Interest

IH, KD, and MK are employees of TNH Health. TNH Health created the chatbot and paid for the cost of submitting the publication. The remaining authors declare that the research was conducted in the absence of any commercial or financial relationships that could be construed as a potential conflict of interest.
